# Cytosolic and nuclear caspase-8 have opposite impact on survival after liver resection for hepatocellular carcinoma

**DOI:** 10.1186/1471-2407-13-532

**Published:** 2013-11-09

**Authors:** Ronald Koschny, Sylvia Brost, Ulf Hinz, Jaromir Sykora, Emanuela M Batke, Stephan Singer, Kai Breuhahn, Wolfgang Stremmel, Henning Walczak, Peter Schemmer, Peter Schirmacher, Tom M Ganten

**Affiliations:** 1 Department of Gastroenterology, University Hospital Heidelberg, Im Neuenheimer Feld 410, 69120, Heidelberg, Germany; 2 Department of General and Transplant Surgery, University Hospital Heidelberg, Heidelberg, Germany; 3 Institute of Pathology, University Hospital Heidelberg, Heidelberg, Germany; 4 Centre for Cell Death, Cancer and Inflammation (CCCI), UCL Cancer Institute, University College London, London, UK

**Keywords:** HCC, Apoptosis, TRAIL receptors, Nuclear caspase-8

## Abstract

**Background:**

An imbalance between proliferation and apoptosis is one of the main features of carcinogenesis. TRAIL (TNF-related apoptosis-inducing ligand) induces apoptosis upon binding to the TRAIL death receptors, TRAIL receptor 1 (TRAIL-R1) and TRAIL-R2, whereas binding to TRAIL-R3 and TRAIL-R4 might promote cell survival and proliferation. The anti-tumor activity of TRAIL-R1 and TRAIL-R2 agonists is currently investigated in clinical trials. To gain further insight into the regulation of apoptosis in hepatocellular carcinoma (HCC), we investigated the TRAIL pathway and the regulators of apoptosis caspase-8, Bcl-xL and Mcl-1 in patients with HCC regarding patient survival.

**Methods:**

We analyzed 157 hepatocellular carcinoma patients who underwent partial liver resection or orthotopic liver transplantation and healthy control liver tissue using immunohistochemistry on tissue microarrays for the expression of TRAIL-R1 to TRAIL-R4, caspase-8, Bcl-xL and Mcl-1. Immunohistochemical data were evaluated for potential associations with clinico-pathological parameters and survival.

**Results:**

Whereas TRAIL-R1 was downregulated in HCC in comparison to normal liver tissue, TRAIL-R2 and –R4 were upregulated in HCC, especially in G2 and G3 tumors. TRAIL-R1 downregulation and upregulation of TRAIL-R2 and TRAIL-R4 correlated with tumor dedifferentiation (G2/G3). TRAIL-R3, Bcl-xL and Mcl-1 showed no differential expression in tumor tissue compared to normal tissue. The expression levels of TRAIL receptors did not correlate with patient survival after partial hepatectomy. Interestingly, in tumor tissue, but not in normal hepatocytes, caspase-8 showed a strong nuclear staining. Low cytosolic and high nuclear staining intensity of caspase-8 significantly correlated with impaired survival after partial hepatectomy, which, for cytosolic caspase-8, was independent from tumor grade.

**Conclusions:**

Assessment of TRAIL-receptor expression patterns may have therapeutic implications for the use of TRAIL receptor agonists in HCC therapy. Tumor-specific nuclear localisation of caspase-8 in HCC suggests an apoptosis-independent function of caspase-8 and correlates with patient survival.

## Background

Hepatocellular carcinoma (HCC) is the main type of primary liver cancer and the fifth most common malignant cancer worldwide. Its poor prognosis makes it the third leading cause of cancer-related mortality [1-3]. Only about 30% of patients are eligible for curative therapies (e.g. resection and transplantation) and the disease recurs frequently following liver resection [4]. Sorafenib, an oral multikinase inhibitor, is effective in the treatment of advanced HCC [5]. However, sorafenib therapy is limited by side effects and lack of long-term efficacy.

The tumor necrosis factor (TNF)-related apoptosis inducing-ligand (TRAIL) is a member of the TNF cytokine family. TRAIL is currently in clinical development as a potential novel anticancer therapeutic because it selectively induces apoptosis in cancer cells [6-11]. After TRAIL-binding TRAIL-R1, also called Death Receptor 4 (DR4), [12] and TRAIL-R2 (DR5) [13,14] initiate apoptosis following formation of the death-inducing signaling complex (DISC): trimerization of TRAIL-R1 and/or TRAIL-R2 leads to recruitment of FADD and cytoplasmic caspase-8 to the intracellular death domain (DD) of both receptors. Caspase-8 recruitment to the DISC activates this protease, which triggers a caspase cascade and, ultimately, apoptotic death of susceptible cells. Two other receptors, TRAIL-R3 and TRAIL-R4, do not induce apoptosis; they lack a functional intracellular death domain [15-17] and have been suggested to inhibit TRAIL-induced apoptosis by competing with TRAIL-R1 and TRAIL-R2 for TRAIL-binding. TRAIL-R4 has also been shown to inhibit apoptosis through ligand-independent association with TRAIL-R2 via the preligand assembly domain (PLAD) [18] or by NF-κB activation upon TRAIL-R4 overexpression [17]. The fifth TRAIL-receptor, osteoprotegerin, is a soluble receptor and is mainly involved in the regulation of bone metabolism [19,20].

Apart from representing potential therapeutic targets for novel, TRAIL-based therapies, the two TRAIL receptors and their expression pattern may be both prognostic and predictive for patient survival. However, the currently available data is controversial in this regard. For instance, in renal cell carcinoma high TRAIL-R2 and low TRAIL-R4 expression correlated with poorer overall survival [21]. In breast cancer, expression of TRAIL-R2 was associated with TRAIL-R4 positivity and correlated with poorer survival [22]. In contrast, in colorectal cancer Ullenhag et al. could not detect any correlation of TRAIL-R1 and TRAIL-R2 expression status with patients survival [23].

Caspase-8 is crucial for triggering apoptosis by death receptors since its recruitment to and activation at the DISC is the decisive step for the initiation of the caspase cascade [24]. Besides apoptosis induction non-apoptotic functions of caspase-8 have been discussed, although these non-apoptotic signaling pathways and molecular targets have not been defined yet [25]. Bcl-xL and Mcl-1 belong to the anti-apoptotic B-cell lymphoma-2 (Bcl-2) family of proteins [26]. High expression of Bcl-XL has been associated with more aggressive tumor biology and/or drug resistance to various chemotherapeutic agents in hematologic and solid malignancies [26]. Inhibition of Bcl-xL induces apoptosis and suppresses growth of hepatoma cells in combination with sorafenib [27]. Mcl-1 is overexpressed in about 50% of HCC tissues [28] but on the other hand deletion of Mcl-1 triggers hepatocarcinogenesis in mice [29]. Recent studies have demonstrated that TRAIL expression is altered in HCC in comparison to normal liver tissue, but there are contradictory data about the expression of the different TRAIL receptors in HCC cells and tissues [30-34]. Thus, we analyzed TRAIL receptors and the apoptosis regulatory proteins caspase-8, Bcl-xL and Mcl-1 in correlation with HCC grading and survival.

## Methods

### Patient characteristics

To analyze the expression of TRAIL receptors, caspase-8, Bcl-xL and Mcl-1 HCC tumor samples were obtained from patients with HCC (n = 157) who underwent orthotopic liver transplantation (OLT, n = 82, 52%) or partial resection (PR, n = 75, 48%) between 1997 and 2005. Median age of the patients was 58 years. 27% suffered from alcohol-induced liver disease, 40% had chronic viral hepatitis. 41 (55%) of the patients undergoing partial liver resection suffered from cirrhosis. Detailed patient characteristics are shown in Table 1.

**Table 1:** Patient’s characteristics

**Type of surgery**	**OLT**	**PR**	**Cohort A**	**Cohort B**
			**all**	**PR for survival analysis**
n = 157	82 (52%)	75 (48%)	157	49
**Median age**			57.6	64.1
Male	68 (83%)	63 (84%)	131 (83%)	42 (86%)
Female	14 (17%)	12 (16%)	26 (17%)	7 (14%)
**Cirrhosis** (histologically confirmed)	70 (85%)	41 (55%)	111 (71%)	24 (49%)
**Etiology:**				
- Alcohol-induced liver disease	29 (35.4%)	13 (17.3%)	42 (27%)	8 (16%)
- Viral disease	38 (46%)	19 (25%)	63 (40%)	9 (18%)
∎ HBV	15 (18.3%)	9 (12%)	24 (15.2%)	5 (10%)
∎ HCV	26 (31.7)	7 (9.3%)	33 (21%)	5 (10%)
∎ HBV + HCV	3 (3.7%)	3 (4%)	6 (3.8)	1 (2%)
- Others (cryptogenic, hemochromatosis, AIH, PBC)	9 (11%)	43 (57%)	52 (33%)	21 (43%)
**TNM**				
pT1	11 (13%)	13 (17%)	24 (15%)	10 (20%)
pT2	30 (37%)	34 (45%)	64 (41%)	23 (47%)
pT3	11 (13%)	17 (23%)	28 (18%)	9 (18%)
pT4	17 (21%)	5 (7%)	22 (14%)	3 (6%)
pTx	13 (16%)	6 (8%)	19 (12%)	4 (8%)
pN0	40 (49%)	27 (36%)	67 (43%)	20 (41%)
pNx	40 (49%)	47 (63%)	87 (55%)	28 (57%)
pN1	2 (2%)	1 (1%)	3 (2%)	1 (2%)
pM0/Mx	80 (98%)	72 (96%)	152 (97%)	48 (98%)
pM1	2 (2%)	3 (4%)	5 (3%)	1 (2%)
**Grading**				
G1	9 (11%)	6 (8%)	15 (10%)	6 (12%)
G2	45 (55%)	42 (56%)	87 (55%)	27 (55%)
G3	28 (34%)	26 (35%)	54 (34%)	16 (33%)
G4	-	1 (1%)	1 (1%)	-

Survival analysis was carried out in 49 patients who underwent partial resection. Of the 75 patients who underwent partial resection, seven were excluded from the survival analysis because they died within the first month after surgery, not related to HCC; 19 patients were lost during follow up. OLT patients were excluded from survival analysis since survival after OLT is mainly influenced by non-tumor-related factors.

### Histopathological data

Normal liver tissue was obtained from liver resections from patients without underlying liver disease who underwent resection for other reasons than HCC, i.e. liver metastasis. All specimens were resected at the Dept. of General and Transplant Surgery, University of Heidelberg. Histopathological data were obtained from the Institute of Pathology, University Hospital of Heidelberg and were reviewed by at least two board-certified pathologists experienced in liver pathology. A total of 157 human liver tissue samples were evaluated by tissue microarrays (TMAs). TMAs contained two representative areas of hepatocellular carcinoma of each patient or normal liver (punch cylinder diameter: 0.6 mm). All specimens were fixed in 4% formalin (pH 7.4) and embedded in paraffin. Grading was determined based on the AFIP system [35]. The study was approved by the ethics committee of the medical faculty of Heidelberg University (206/2005).

### Antibodies and reagents

For specific immunohistochemical detection of TRAIL receptors we used the following mouse IgG antibodies as described before [22]: TR1.02 (TRAIL-R1; mIgG2b), TR2.21 (TRAIL-R2; mIgG1), TR3.06 (TRAIL-R3; mIgG1) and TR4.18 (TRAIL-R4; mIgG1). The antibody C-15 (caspase-8, mIgG2b) was kindly provided by P.H. Krammer (DKFZ, Heidelberg). Furthermore, the following antibodies were used: 2H212 (Bcl-xL, mIgG2a, Zytomed, Berlin, Germany), S-19 (Mcl-1, rabbit polyclonal IgG, Santa Cruz, Santa Cruz, CA, USA), C92-605 (active caspase-3, BD Biosciences, San Jose, USA), 18C8 (active caspase-8, Cell Signalling, Danvers, MA, USA). Super-Sensitive Detection Kit from BioGenex (Fremont, CA, USA) was used for detection. The specificity of immunohistochemical stainings of the different anti-TRAIL-R mAbs was determined by staining of sections of formalin-fixed, paraffin-embedded pellets of CV1 cells transfected with pCDNA3.1-based expression vectors for TRAIL-R1 to TRAIL-R4 as described previously [22]. For TRAIL-R1 staining with TR1.02, a high temperature antigen retrieval step was performed by incubating in 10 mM citrate buffer (Target Retrieval Solution, S1699 DakoCytomation, Glostrup, Denmark) at pH 6.0 at 89°C for 15 min. For paraffin sections stained for TRAIL-R2 with TR2.21, TRAIL-R3 with TR3.06 and TRAIL-R4 with TR4.18, antigen retrieval was achieved by incubation in 10 mM citrate buffer (pH 6.0) at 99°C for 25 min. For Bcl-xL antigen demasking was performed by incubation in EDTA (1 mM, pH 8.0) at 99°C for 15 min. For staining with the other antibodies antigen demasking was performed in 10 mM citrate buffer (pH 6.0) at 99°C for 25 min. To block non-specific antibody binding, sections were incubated with blocking solution 1 (PBS, BSA 20 mg/ml (Serva, Heidelberg, Germany), human IgG 1 mg/ml Gamma-Venin, (Aventis Behring, Marburg, Deutschland)) for 20 min. Sections were then incubated in the presence of the first antibody at room temperature for one hour (caspase-8: 10 μg/ml, Bcl-xL: 7 μg/ml, Mcl-1: 0,5 μg/ml) or at 4°C in blocking solution overnight (TRAIL-R1: 20 μg/ml, TRAIL-R2: 5 μg/ml, TRAIL-R3: 10 μg/ml, TRAIL-R4: 5 μg/ml) or isotype-matched antibodies (IgG1 or IgG2b) at the same concentration, both obtained from DakoCytomation. Sections were washed twice in PBS and incubated with blocking solution 2 (20% normal goat serum from Jackson ImmunoResearch, West Grove, PA, USA) for 20 min. After blocking, sections were incubated with secondary biotinylated antibody at room temperature for 30 min, rinsed twice for 5 min in PBS and incubated for 30 min with streptavidin-alkaline phosphatase [Super-Sensitive Detection Kit, BioGenex]. Thereafter, sections were rinsed twice in PBS, incubated with fast red substrate (Fast Red Substrate System, DakoCytomation) and counterstained with haematoxylin (DakoCytomation).

### Histopathological scoring and statistics

A two-dimensional scoring system was applied to semi-quantitatively assess the expression of the respective protein. The percentage of positive cells was estimated by two independent investigators on a scale from 0 to 100% and categorised from 0–4 (0 = 0, 1 = up to 1%, 2 = 1-10%, 3 = 10-50%, 4 = more than 50%). Intensity of staining (intensity score) was judged on an arbitrary scale of 0 to 4: no staining (0), weak positive staining (1), moderate positive staining (2), strong positive staining (3) and very strong positive staining (4) as described by Zhuang et al. [36] and applied to tumor tissue arrays [22]. The immunoreactive score (IRS) was calculated by multiplying the percentage of positive cells (0–4, according to the categorised percentages of positive cells) with staining intensity (0–4, according to the category of staining intensity). For instance, a tumor sample with 60% of positive tumor cells (=category 4 for “percentage of positive cells”) with a very strong staining (=category 4 for “staining intensity”) will result in an IRS of 4 × 4 = 16, which represents the maximum IRS. From each tumor, two samples were spotted and analyzed on the tissue microarray (TMA). The final IRS of each patient was calculated as the mean of the two investigator’s analyses of both tumor samples.

Statistical analysis was performed using SAS software (Release 9.1, SAS Institute, Cary, NC). The nonparametric Mann–Whitney *U*-test was used to compare the immunohistochemical score of TRAIL receptors and key proteins between tumor and normal liver tissue and graphically represented in Box plots. Comparisons of the immunohistochemical score between more than two subgroups were performed using the Kruskal-Wallis test. Linear correlation between nuclear/cytosolic caspase-8 expression and Ki67/apoptosis rate was performed using the Spearman correlation coefficient and the corresponding p-value. Overall survival was defined as the time from the date of tumor staging to either death from any cause or last follow-up. Patients alive at the last follow-up were censored. Survival curves were constructed by using the Kaplan-Meier estimate. The 1-, 2-, 5-, and 10-year survival rates and the median survival time are presented. Differences between survival curves of subgroups of patients were analyzed by the log-rank test. The immunohistochemical scores for TRAIL-R1 to TRAIL-R4 and caspase-8 were dichotomized for survival analysis according to the quartiles and the median of the distribution of the score values to ensure a sufficient number of patients in the resulting subgroups. The Cox proportional hazards regression analysis was used to analyze the correlation of survival and expression of TRAIL receptors, other key proteins, and histopathological parameters. Two sided p values were always computed and p values < 0.05 were considered statistically significant.

## Results

**Figure 1:**
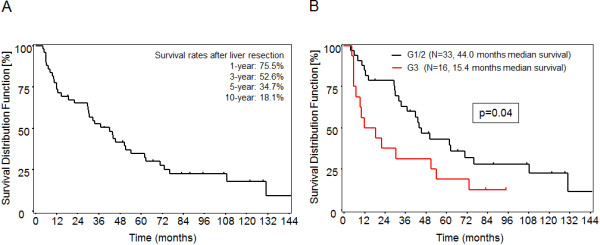
**Survival rates of HCC patients after partial liver resection. A**: Survival rates of HCC patients who underwent partial liver resection using the Kaplan-Meier estimate. **B**: Survival rates in HCC patients with G3 tumors compared to G1 and G2 tumors.

We compared the expression profile of TRAIL-R1 to TRAIL-R4, caspase-8, Bcl-xL and Mcl-1 in HCC in comparison to normal liver tissue. All TRAIL receptors showed both cytoplasmic and membranous staining, although membrane staining was rather faint and therefore not quantified. Survival in correlation with the immunohistochemical staining result was analyzed in 49 patients who underwent partial liver resection (see Material and Methods). Overall survival rates of these patients were 75.5%, 52.6%, 34.7% and 18.1% after one, three, five, and ten years, respectively. Median survival was 42 months (Figure 1A). Survival rates were poorer in patients with G3 tumors compared to G1 and G2 tumors (Figure 1B).

**Figure 2:**
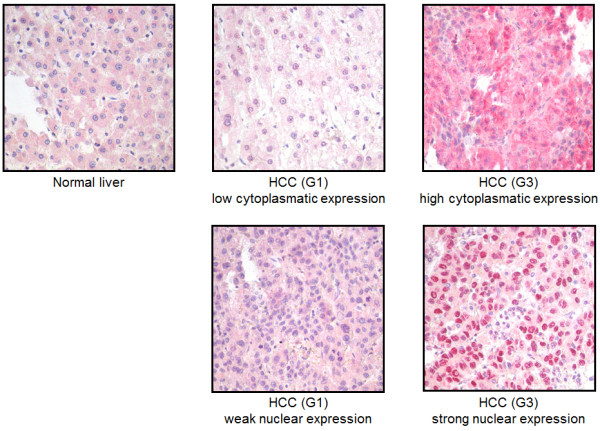
**Cytosolic and nuclear expression of caspase-8.** Immunohistochemical staining of caspase-8 (red staining) in healthy liver and HCC (G1 versus G3) with cytosolic or nuclear localisation (20 × magnified).

Investigating the expression level of caspase-8, we found a differential expression of caspase-8 in the cytosol versus nucleus of tumor cells (examples presented in Figure 2). Thus, the cytosolic and nuclear expression patterns of caspase-8 were analyzed separately in the subsequent investigations.

### Expression levels of TRAIL-R1, TRAIL-R2, TRAIL-R4 and nuclear caspase-8 correlate with the grade of malignancy of HCCs

To analyse the expression level of important regulators of TRAIL-induced apoptosis, tumor tissues from explanted livers (OLT) and partial liver resections (PR) were used in a tissue microarray (cohort A, Table 1, n = 157).

**Figure 3:**
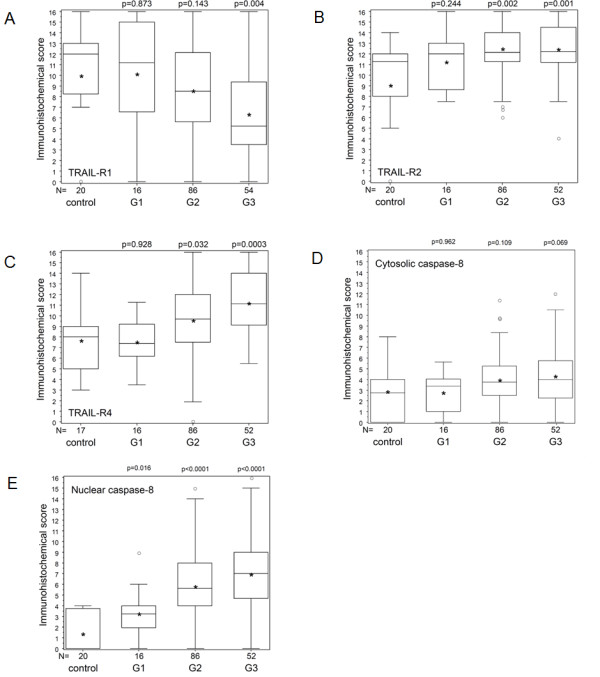
**Protein expression levels and WHO grade of malignancies.** Expression of TRAIL-R1 **(A)**, TRAIL-R2 **(B)**, TRAIL-R4 **(C)**, cytosolic caspase-8 **(D)**, and nuclear caspase-8 **(E)** expression in HCC specimens according to tumor grading compared to normal liver tissue. Statistical analysis was performed by the Wilcoxon test. Statistical significance is indicated for all tumor grades in comparison to healthy liver controls.

The death-inducing receptors TRAIL-R1 and TRAIL-R2 were differentially expressed in normal liver versus HCC. TRAIL-R1 showed strong cytoplasmic staining in normal liver tissue with a mild downregulation in G1 and G2 tumors but a significant downregulation in G3 tumors compared to normal tissue (p = 0.004), but also compared to G1 (p = 0.01) and G2 (p = 0.003) tumors (Figure 3A). In contrast, TRAIL-R2 was expressed in normal liver tissue and significantly upregulated in G2 (p = 0.002) and G3 (p = 0.001) tumor tissue compared to normal tissue (Figure 3B). TRAIL-R2 expression did not significantly differ between G2 and G3 (p = 0.69) or between G1 and G3 (p = 0.07) tumors. TRAIL-R3 showed only low expression, both in normal liver tissue and tumor tissue, which did not correlate with tumor grade (data not shown). TRAIL-R4 was moderately expressed in normal liver tissue and significantly upregulated in G2 (p = 0.032) and G3 (p = 0.0003) tumors (Figure 3C). TRAIL-R4 expression in G3 tumors also significantly differed from G1 (p < 0.001) and G2 (p = 0.012) tumors. Caspase-8 showed only a low level expression in the cytoplasm of normal hepatocytes (Figure 2A), which did not significantly differ from cytosolic expression of caspase-8 in HCCs (Figure 3D). Although in normal liver tissue caspase-8 could not be detected in the nucleus, many HCC samples showed nuclear expression of caspase-8 (Figure 2). Nuclear caspase-8 was significantly higher expressed in G1 (p = 0.016), G2 (p < 0.0001), and G3 (p < 0.0001) HCCs compared to normal liver tissue (Figure 3E). G3 tumors also demonstrated a significantly higher expression of nuclear caspase-8 compared to G1 (p = 0.001) but not compared to G2 (p = 0.06) HCC tissues.

### Cytosolic and nuclear caspase-8 expression levels correlate with survival after partial liver resection

The correlation between survival and protein expression was analysed in n = 49 HCC patients undergoing partial liver resection, for whom survival data were available (Table 1, Cohort B). The correlation between TRAIL-receptor and caspase-8 expression levels and tumor grade showed identical levels of significance in this smaller subgroup.

**Figure 4:**
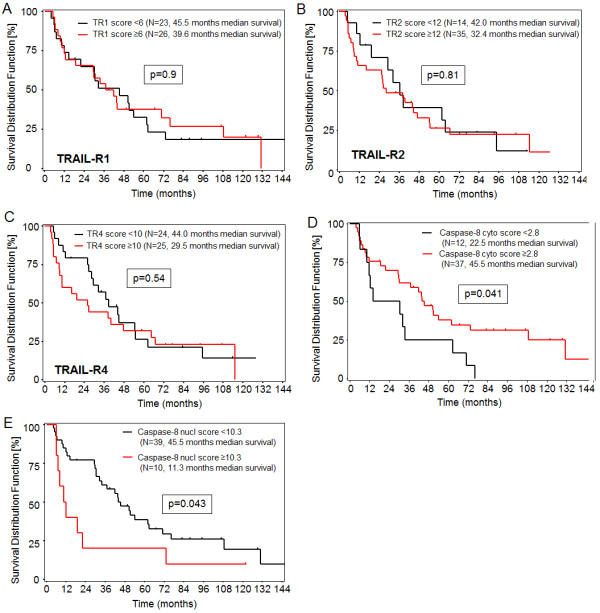
**Protein levels and patient’s survival rates.** Overall survival after partial resection for HCC (n = 49) according to expression of TRAIL-R1 **(A)**, TRAIL-R2 **(B)**, TRAIL-R4 **(C)**, cytosolic caspase-8 **(D)**, and nuclear caspase-8 **(E)** was calculated by the Kaplan-Meier estimate. Thresholds for high and low protein expression are given for each protein.

Neither TRAIL-R1, TRAIL-R2 nor TRAIL-R4 expression scores correlated with patient survival (Figure 4A-C). However, high cytosolic caspase-8 expression in tumor tissue (IRS ≥2.8) significantly correlated with better survival (Figure 4D). Multivariate Cox regression analysis confirmed cytosolic caspase-8 to be a survival predictor independent from tumor grading [G3 versus G1/2: HR = 2.28 (95% CI: 1.14-4.55), p = 0.0196 and cytosolic caspase-8 <2.8 versus ≥2.8: HR = 2.39 (95% CI: 1.16-4.92), p = 0.0182]. In contrast, high nuclear expression of caspase-8 (IRS >10.3) was associated with shorter survival rates in patients after partial liver resection (Figure 4E). Because of the strong correlation of nuclear expression of caspase-8 and tumor grading and the low number of patients with nuclear caspase-8 IRS ≥10.3, none of the two factors were significantly associated with survival in a multivariate Cox regression analysis [G3 vs G1/2: HR = 1.72 (95% CI: 0.85-3.49, p = 0.134 and nuclear caspase-8 ≥10.3 versus <10.3: HR = 1.80 (95% CI: 0.81-4.01)].

## Discussion

In this study we assessed the expression of TRAIL receptors, caspase-8, Bcl-xL and Mcl-1 in 157 patients with hepatocellular carcinoma and normal liver tissue using tissue microarrays, and correlated the expression with clinico-pathological parameters. Survival analysis was carried out for patients who underwent liver resection.

TRAIL-R1 was significantly downregulated in less differentiated HCC. However, TRAIL-R1 expression did not correlate with patient survival after liver resection. Kriegl et al. reported a significant membrane staining of TRAIL-R1 in HCC compared to normal liver tissue and a longer survival of HCC patients undergoing partial hepatectomy with TRAIL-R1 membrane positive versus negative tumors [31]. However, our immunohistochemical analysis detected considerable cytoplasmic but not membrane TRAIL-R1 staining [31,37]. Having established the specificity of our TRAIL-R1 (and TRAIL-R2) antibodies in TRAIL-R1- (and TRAIL-R2-) transfected cells, cytoplasmic staining prevailed also in this setting [22]. Using the highly specific antibodies for TRAIL-R1 and TRAIL-R2, HCC cell lines also displayed strong cytoplasmic, rather than membrane, staining which was confirmed by flow cytometry (data not shown). Upon TRAIL death receptor upregulation by chemotherapeutic drugs, membrane staining of both receptors could be detected in HCC cell lines which was paralleled by enhanced surface receptor as detected by flow cytometry [10]. These control experiments support the sensitivity and high specificity of our TRAIL receptor antibodies for both cytoplasmic and membrane staining. Our data are in line with reports on strong cytoplasmic rather than membrane staining of both TRAIL-R1 and TRAIL-R2 in primary HCC tissue [33]. Correlation analyses of TRAIL-R1 expression and survival in other tumor entities revealed contradictory results. In colorectal cancer both low [38] and high [39] TRAIL-R1 expression correlated with poorer survival. Ullenhag et al. found no correlation between TRAIL-R1 expression level and survival in colorectal cancer patients [23].

In our study both TRAIL-R2 and TRAIL-R4 were upregulated in dedifferentiated HCCs. However, for none of the TRAIL receptors expression correlated with patient survival. In previous studies high expression of TRAIL-2 [40] was also associated with less differentiated tumors and implied poorer survival in breast cancer [22,41], renal cell carcinoma [21], and NSCLC [40]. In the report by Kriegl et al., TRAIL-R2 membrane staining correlated with better survival of HCC patients after partial liver resection [31]. However, as stated above, in our cohort no relevant TRAIL-R2 membrane staining could be detected in HCC tissues. In summary, TRAIL receptor expression patterns seem to vary between different tumor entities and, therefore, their correlation with survival data may depend on tumor type and clinical setting (adjuvant, curative and palliative treatment).

Downregulation of TRAIL-R2 *in vivo* may mirror the selection pressure by antitumor immune responses (e.g. by TRAIL-expressing NK cells). On the other hand, TRAIL-R2-positive tumor cells may have developed TRAIL resistance downstream of the receptor level, thereby allowing for tumor cell proliferation despite TRAIL death receptor expression. Nevertheless, many chemotherapeutic drugs sensitize resistant tumor cells to TRAIL-induced apoptosis via enhancement of proapoptotic regulators of the extrinsic and intrinsic pathway [8,10,42]. Thus, HCCs with high TRAIL-R2 expression should be eligible for combinatorial TRAIL-based therapies. Previously, we could show that TRAIL-R2 expression was highly correlated with TRAIL-R4 positivity in breast cancer [22]. TRAIL-R4 overexpression correlated with poorer survival in breast [22] and prostate cancer [43]. Applying TRAIL-R2-specific agonists (e.g. the TRAIL-R2-specific antibody lexatumumab) may bypass the anti-apoptotic effects of high TRAIL-R4 expression and allow for effective tumor treatment [11]. It has been discussed that therapeutic implications of TRAIL-based therapies might be limited by toxicity to non-transformed human hepatocytes [44,45]. Yet, we previously showed that there is a large therapeutic window which allows effective TRAIL-based cancer therapy [10].

Analysis of the two anti-apoptotic Bcl-2 family members Bcl-xL and Mcl-1 revealed low expression of Bcl-xL in normal liver tissue, which was not-significantly upregulated in G2 and G3 tumors (data not shown). Expression of Mcl-1 was also increased in G3 tumors as compared to G1/2 tumors and normal tissue; however no correlation with survival could be detected (data not shown).

As the main initiator caspase of the TRAIL pathway, caspase-8 is located in the cytosol to be recruited to the TRAIL DISC after ligand binding to TRAIL-R1/R2. Loss or downregulation of caspase-8 has been proposed as a possible mechanism of apoptosis resistance in tumor cells [46]. In our cohort, high cytosolic caspase-8 expression correlated with better survival independently from tumor grade, possibly reflecting the higher apoptotic potential of these tumor cells. Interestingly, we could demonstrate nuclear staining of caspase-8 in HCCs but not in normal hepatocytes. The staining intensity of nuclear caspase-8 correlated with grade of malignancy but also with poorer patient survival. Due to the strong correlation between nuclear expression of caspase-8 and tumor grading, multivariate Cox regression analysis could not detect an influence of nuclear caspase-8 on survival independent from the tumor grade. However, patient number with a nuclear caspase-8 score ≥10.3 might be too small (n = 10) for a multivariate analysis of the two parameters, high nuclear caspase-8 and tumor grading. Thus, our data need to be scrutinized in a larger cohort. Although high nuclear and cytosolic caspase-8 expression have an opposed effect on patient survival, high nuclear and cytoplasmic caspase-8 expression is not mutually exclusive, since 9 out of 56 patients (16%) and 3 out of 14 patients (21%) with a high nuclear caspase-8 score of ≥7 and ≥10.3, respectively, had also an equally high cytoplasmic caspase-8 expression level. Most of these patients had WHO grade 3 tumors (78% for a score ≥7, 100% for a score of ≥10).

Whereas the role of cytosolic caspase-8 as a factor in triggering apoptosis via death receptors has been well examined [24,47,48], nuclear translocation of caspase-8 has so far not been described in HCCs. In contrast, nuclear localisation of caspase-8 has been found in apoptotic neurons [49]. Since these cells were undergoing apoptosis, caspase-8 was suspected to shuttle to the nucleus to exert cleavage of the DNA repair enzyme PARP2, a hallmark of apoptotic cell death. In contrast to apoptotic neurons, in our study nuclear caspase-8 was detected in nearly all tumor cells of poorly differentiated HCCs (Figures 2 and 3E) and nuclear caspase-8 expression did not correlate with the apoptosis rate (r = 0.078, p = 0.420). This may indicate a non-apoptotic function of caspase-8 in HCCs. Enhancement of tumor cell migration and inhibition of Fas-induced apoptosis has been recently described as a non-apoptotic function of caspase-8 in different experimental cancer cell lines, which was not dependent on its catalytic activity but on Src-mediated phosphorylation of Tyr380 in a linker region between the small and large caspase-8 subunits [50,51]. Metastasis formation of non-apoptotic neuroblastoma cells was enhanced by recruitment of caspase-8 to the cellular migration machinery [52]. Interestingly, in our cohort, high nuclear expression of caspase-8 correlated with a higher proliferation index of tumor cells (Ki67, r = 0.282, p = 0.0004, whereas the cytosolic expression of caspase-8 did not (r = 0.089, p = 0.274). A recent study has shown that caspase-8 can be sumoylated at lysine 156 leading to a 75 kDa isoform (p75) and that sumoylation of caspase-8 by SUMO-1 is associated with nuclear localization of caspase-8 [53] suggesting that nuclear expression of caspase-8 in our study might be a result of sumoylation. Interestingly, SUMO-1 is overexpressed in HCCs [54] and expression profiling has shown that HCC patients with shorter survival show higher expression of genes involved in sumoylation [55,56]. Although the physiological relevance of sumoylated caspase-8 is unclear, recent studies suggest that sumoylation of caspase-8 does not impair cytoplasmic caspase-8 activation, but that sumoylated nuclear caspase-8 (p75) can presumably cleave other, so far undefined, specific nuclear substrates [53]. However, using a cleavage-specific antibody for caspase-8, we could not detect activated caspase-8 in the nuclei of tumor cells in our cohort.

## Conclusions

In conclusion, differential expression of TRAIL-R1, TRAIL-R2 and TRAIL-R4 may help to histopathologically identify hepatocellular carcinoma patients who could benefit from TRAIL-based therapies. Prospective studies are needed to confirm the predictive role of TRAIL-receptor expression patterns for TRAIL-based therapies or TRAIL-dependent mechanisms of other chemotherapeutic drugs. Furthermore, the prognostic role of nuclear localisation of caspase-8 needs to be confirmed in larger trials and other tumor entities. Identifying the molecular targets and pathophysiological consequences of nuclear caspase-8 may reveal novel, non-apoptotic functions of this crucial initiator caspase.

## Abbreviations

Bcl-xL: B-cell lymphoma-extra large; DR: Death receptor; HCC: Hepatocellular carcinoma; Mcl-1: Induced myeloid leukemia cell differentiation protein; mIgG: Mouse IgG; NF-κB: Nuclear factor kappa B; OLT: Orthotopic liver transplantation; PLAD: Preligand assembly domain; SUMO: Small Ubiquitin-like Modifier; TNF: Tumor necrosis factor; TRAIL: TNF-related apoptosis inducing ligand; TRAIL-R1: TRAIL-receptor 1; TRAIL-R2: TRAIL-receptor 2; TRAIL-R3: TRAIL-receptor 3; TRAIL-R4: TRAIL-receptor 4.

## Competing interests

The authors declare that they have no competing interests.

## Authors’ contributions

SB and RK participated in the design of the study and wrote the manuscript together with TMG. JS established the staining protocols for all antibodies and carried out the immunohistochemical studies together with SB and EB. EB collected clinical data. UH performed the statistical analysis. PS participated in the design and coordination of the study. WS conceived of the study, and participated in its design and coordination and helped to draft the manuscript. PS, SS and KB provided the histoarrays and revised the manuscript. HW developed all TRAIL-receptor-specific antibodies employed in this study, oversaw the establishing of the staining protocols for all antibodies, initiated and designed the study together with TMG, and revised the manuscript. All authors read and approved the final manuscript.

## Pre-publication history

The pre-publication history for this paper can be accessed here:

http://www.biomedcentral.com/1471-2407/13/532/prepub

## References

[B1] Venook AP Papandreou C Furuse J de Guevara LL The incidence and epidemiology of hepatocellular carcinoma: a global and regional perspective Oncologist 2010 15 Suppl 4 5 13 21115576 10.1634/theoncologist.2010-S4-05

[B2] Parkin DM Bray F Ferlay J Pisani P Global cancer statistics, 2002 CA Cancer J Clin 2005 55 2 74 108 10.3322/canjclin.55.2.74 15761078

[B3] Breuhahn K Gores G Schirmacher P Strategies for hepatocellular carcinoma therapy and diagnostics: lessons learned from high throughput and profiling approaches Hepatology 2011 53 6 2112 2121 10.1002/hep.24313 21433041

[B4] Koschny R Schmidt J Ganten TM Beyond Milan criteria–chances and risks of expanding transplantation criteria for HCC patients with liver cirrhosis Clin Transplant 2009 23 Suppl 21 49 60 19930317 10.1111/j.1399-0012.2009.01110.x

[B5] Llovet JM Ricci S Mazzaferro V Hilgard P Gane E Blanc JF de Oliveira AC Santoro A Raoul JL Forner A Sorafenib in advanced hepatocellular carcinoma N Engl J Med 2008 359 4 378 390 10.1056/NEJMoa0708857 18650514

[B6] Wiley SR Schooley K Smolak PJ Din WS Huang CP Nicholl JK Sutherland GR Smith TD Rauch C Smith CA Identification and characterization of a new member of the TNF family that induces apoptosis Immunity 1995 3 6 673 682 10.1016/1074-7613(95)90057-8 8777713

[B7] Pitti RM Marsters SA Ruppert S Donahue CJ Moore A Ashkenazi A Induction of apoptosis by Apo-2 ligand, a new member of the tumor necrosis factor cytokine family J Biol Chem 1996 271 22 12687 12690 10.1074/jbc.271.22.12687 8663110

[B8] Ganten TM Koschny R Haas TL Sykora J Li-Weber M Herzer K Walczak H Proteasome inhibition sensitizes hepatocellular carcinoma cells, but not human hepatocytes, to TRAIL Hepatology 2005 42 3 588 597 10.1002/hep.20807 16037944

[B9] Ganten TM Koschny R Sykora J Schulze-Bergkamen H Buchler P Haas TL Schader MB Untergasser A Stremmel W Walczak H Preclinical differentiation between apparently safe and potentially hepatotoxic applications of TRAIL either alone or in combination with chemotherapeutic drugs Clin Cancer Res 2006 12 8 2640 2646 10.1158/1078-0432.CCR-05-2635 16638878

[B10] Koschny R Ganten TM Sykora J Haas TL Sprick MR Kolb A Stremmel W Walczak H TRAIL/bortezomib cotreatment is potentially hepatotoxic but induces cancer-specific apoptosis within a therapeutic window Hepatology 2007 45 3 649 658 10.1002/hep.21555 17326159

[B11] Koschny R Walczak H Ganten TM The promise of TRAIL-potential and risks of a novel anticancer therapy J Mol Med 2007 85 9 923 935 10.1007/s00109-007-0194-1 17437073

[B12] Pan G O’Rourke K Chinnaiyan AM Gentz R Ebner R Ni J Dixit VM The receptor for the cytotoxic ligand TRAIL Science 1997 276 5309 111 113 10.1126/science.276.5309.111 9082980

[B13] Walczak H Degli-Esposti MA Johnson RS Smolak PJ Waugh JY Boiani N Timour MS Gerhart MJ Schooley KA Smith CA TRAIL-R2: a novel apoptosis-mediating receptor for TRAIL Embo J 1997 16 17 5386 5397 10.1093/emboj/16.17.5386 9311998 PMC1170170

[B14] Sheridan JP Marsters SA Pitti RM Gurney A Skubatch M Baldwin D Ramakrishnan L Gray CL Baker K Wood WI Control of TRAIL-induced apoptosis by a family of signaling and decoy receptors Science 1997 277 5327 818 821 10.1126/science.277.5327.818 9242611

[B15] Pan G Ni J Wei YF Yu G Gentz R Dixit VM An antagonist decoy receptor and a death domain-containing receptor for TRAIL Science 1997 277 5327 815 818 10.1126/science.277.5327.815 9242610

[B16] Mongkolsapaya J Cowper AE Xu XN Morris G McMichael AJ Bell JI Screaton GR Lymphocyte inhibitor of TRAIL (TNF-related apoptosis-inducing ligand): a new receptor protecting lymphocytes from the death ligand TRAIL J Immunol 1998 160 1 3 6 9551946

[B17] Degli-Esposti MA Dougall WC Smolak PJ Waugh JY Smith CA Goodwin RG The novel receptor TRAIL-R4 induces NF-kappaB and protects against TRAIL-mediated apoptosis, yet retains an incomplete death domain Immunity 1997 7 6 813 820 10.1016/S1074-7613(00)80399-4 9430226

[B18] Clancy L Mruk K Archer K Woelfel M Mongkolsapaya J Screaton G Lenardo MJ Chan FK Preligand assembly domain-mediated ligand-independent association between TRAIL receptor 4 (TR4) and TR2 regulates TRAIL-induced apoptosis Proc Natl Acad Sci U S A 2005 102 50 18099 18104 10.1073/pnas.0507329102 16319225 PMC1312398

[B19] Simonet WS Lacey DL Dunstan CR Kelley M Chang MS Luthy R Nguyen HQ Wooden S Bennett L Boone T Osteoprotegerin: a novel secreted protein involved in the regulation of bone density [see comments] Cell 1997 89 2 309 319 10.1016/S0092-8674(00)80209-3 9108485

[B20] Falschlehner C Emmerich CH Gerlach B Walczak H TRAIL signalling: Decisions between life and death Int J Biochem Cell Biol 2007 39 7-8 1462 1475 10.1016/j.biocel.2007.02.007 17403612

[B21] Macher-Goeppinger S Aulmann S Tagscherer KE Wagener N Haferkamp A Penzel R Brauckhoff A Hohenfellner M Sykora J Walczak H Prognostic value of tumor necrosis factor-related apoptosis-inducing ligand (TRAIL) and TRAIL receptors in renal cell cancer Clin Cancer Res 2009 15 2 650 659 10.1158/1078-0432.CCR-08-0284 19147771

[B22] Ganten TM Sykora J Koschny R Batke E Aulmann S Mansmann U Stremmel W Sinn HP Walczak H Prognostic significance of tumour necrosis factor-related apoptosis-inducing ligand (TRAIL) receptor expression in patients with breast cancer J Mol Med 2009 87 10 995 1007 10.1007/s00109-009-0510-z 19680616

[B23] Ullenhag GJ Mukherjee A Watson NF Al-Attar AH Scholefield JH Durrant LG Overexpression of FLIPL is an independent marker of poor prognosis in colorectal cancer patients Clin Cancer Res 2007 13 17 5070 5075 10.1158/1078-0432.CCR-06-2547 17785559

[B24] Kantari C Walczak H Caspase-8 and bid: caught in the act between death receptors and mitochondria Biochim Biophys Acta 2011 1813 4 558 563 10.1016/j.bbamcr.2011.01.026 21295084

[B25] Tibbetts MD Zheng L Lenardo MJ The death effector domain protein family: regulators of cellular homeostasis Nat Immunol 2003 4 5 404 409 10.1038/ni0503-404 12719729

[B26] Kang MH Reynolds CP Bcl-2 inhibitors: targeting mitochondrial apoptotic pathways in cancer therapy Clin Cancer Res 2009 15 4 1126 1132 10.1158/1078-0432.CCR-08-0144 19228717 PMC3182268

[B27] Hikita H Takehara T Shimizu S Kodama T Shigekawa M Iwase K Hosui A Miyagi T Tatsumi T Ishida H The Bcl-xL inhibitor, ABT-737, efficiently induces apoptosis and suppresses growth of hepatoma cells in combination with sorafenib Hepatology 2010 52 4 1310 1321 10.1002/hep.23836 20799354

[B28] Sieghart W Losert D Strommer S Cejka D Schmid K Rasoul-Rockenschaub S Bodingbauer M Crevenna R Monia BP Peck-Radosavljevic M Mcl-1 overexpression in hepatocellular carcinoma: a potential target for antisense therapy J Hepatol 2006 44 1 151 157 10.1016/j.jhep.2005.09.010 16289418

[B29] Weber A Boger R Vick B Urbanik T Haybaeck J Zoller S Teufel A Krammer PH Opferman JT Galle PR Hepatocyte-specific deletion of the antiapoptotic protein myeloid cell leukemia-1 triggers proliferation and hepatocarcinogenesis in mice Hepatology 2010 51 4 1226 1236 10.1002/hep.23479 20099303 PMC2936921

[B30] Fabregat I Dysregulation of apoptosis in hepatocellular carcinoma cells World J Gastroenterol 2009 15 5 513 520 10.3748/wjg.15.513 19195051 PMC2653340

[B31] Kriegl L Jung A Engel J Jackstadt R Gerbes AL Gallmeier E Reiche JA Hermeking H Rizzani A Bruns CJ Expression, cellular distribution, and prognostic relevance of TRAIL receptors in hepatocellular carcinoma Clin Cancer Res 2010 16 22 5529 5538 10.1158/1078-0432.CCR-09-3403 20889918

[B32] Shiraki K Yamanaka T Inoue H Kawakita T Enokimura N Okano H Sugimoto K Murata K Nakano T Expression of TNF-related apoptosis-inducing ligand in human hepatocellular carcinoma Int J Oncol 2005 26 5 1273 1281 15809718

[B33] Chen XP He SQ Wang HP Zhao YZ Zhang WG Expression of TNF-related apoptosis-inducing Ligand receptors and antitumor tumor effects of TNF-related apoptosis-inducing Ligand in human hepatocellular carcinoma World J Gastroenterol 2003 9 11 2433 2440 14606071 10.3748/wjg.v9.i11.2433 PMC4656516

[B34] Herr I Schemmer P Buchler MW On the TRAIL to therapeutic intervention in liver disease Hepatology 2007 46 1 266 274 10.1002/hep.21740 17596886

[B35] Nzeako UC Goodman ZD Ishak KG Comparison of tumor pathology with duration of survival of North American patients with hepatocellular carcinoma Cancer 1995 76 4 579 588 10.1002/1097-0142(19950815)76:4<579::AID-CNCR2820760407>3.0.CO;2-D 8625150

[B36] Zhuang L Lee CS Scolyer RA McCarthy SW Zhang XD Thompson JF Hersey P Mcl-1, Bcl-XL and Stat3 expression are associated with progression of melanoma whereas Bcl-2, AP-2 and MITF levels decrease during progression of melanoma Mod Pathol 2007 20 4 416 426 10.1038/modpathol.3800750 17384650

[B37] Zhang Y Zhang B TRAIL resistance of breast cancer cells is associated with constitutive endocytosis of death receptors 4 and 5 Mol Cancer Res 2008 6 12 1861 1871 10.1158/1541-7786.MCR-08-0313 19074831

[B38] Granci V Bibeau F Kramar A Boissiere-Michot F Thezenas S Thirion A Gongora C Martineau P Del Rio M Ychou M Prognostic significance of TRAIL-R1 and TRAIL-R3 expression in metastatic colorectal carcinomas Eur J Cancer 2008 44 15 2312 2318 10.1016/j.ejca.2008.06.042 18755584

[B39] Strater J Hinz U Walczak H Mechtersheimer G Koretz K Herfarth C Moller P Lehnert T Expression of TRAIL and TRAIL Receptors in Colon Carcinoma: TRAIL-R1 Is an Independent Prognostic Parameter Clin Cancer Res 2002 8 12 3734 3740 12473583

[B40] Spierings DC de Vries EG Timens W Groen HJ Boezen HM de Jong S Expression of TRAIL and TRAIL death receptors in stage III non-small cell lung cancer tumors Clin Cancer Res 2003 9 9 3397 3405 12960128

[B41] McCarthy MM Sznol M DiVito KA Camp RL Rimm DL Kluger HM Evaluating the expression and prognostic value of TRAIL-R1 and TRAIL-R2 in breast cancer Clin Cancer Res 2005 11 14 5188 5194 10.1158/1078-0432.CCR-05-0158 16033835

[B42] Ganten TM Haas TL Sykora J Stahl H Sprick MR Fas SC Krueger A Weigand MA Grosse-Wilde A Stremmel W Enhanced caspase-8 recruitment to and activation at the DISC is critical for sensitisation of human hepatocellular carcinoma cells to TRAIL-induced apoptosis by chemotherapeutic drugs Cell Death Differ 2004 11 Suppl 1 S86 S96 15105837 10.1038/sj.cdd.4401437

[B43] Koksal IT Sanlioglu AD Karacay B Griffith TS Sanlioglu S Tumor necrosis factor-related apoptosis inducing ligand-R4 decoy receptor expression is correlated with high Gleason scores, prostate-specific antigen recurrence, and decreased survival in patients with prostate carcinoma Urol Oncol 2008 26 2 158 165 10.1016/j.urolonc.2007.01.022 18312935

[B44] Jo M Kim TH Seol DW Esplen JE Dorko K Billiar TR Strom SC Apoptosis induced in normal human hepatocytes by tumor necrosis factor- related apoptosis-inducing ligand Nat Med 2000 6 5 564 567 10.1038/75045 10802713

[B45] Lawrence D Shahrokh Z Marsters S Achilles K Shih D Mounho B Hillan K Totpal K DeForge L Schow P Differential hepatocyte toxicity of recombinant Apo2L/TRAIL versions Nat Med 2001 7 4 383 385 10.1038/86397 11283636

[B46] Grotzer MA Eggert A Zuzak TJ Janss AJ Marwaha S Wiewrodt BR Ikegaki N Brodeur GM Phillips PC Resistance to TRAIL-induced apoptosis in primitive neuroectodermal brain tumor cells correlates with a loss of caspase-8 expression Oncogene 2000 19 40 4604 4610 10.1038/sj.onc.1203816 11030149

[B47] Muzio M Chinnaiyan AM Kischkel FC O’Rourke K Shevchenko A Ni J Scaffidi C Bretz JD Zhang M Gentz R FLICE, a novel FADD-homologous ICE/CED-3-like protease, is recruited to the CD95 (Fas/APO-1) death–inducing signaling complex Cell 1996 85 6 817 827 10.1016/S0092-8674(00)81266-0 8681377

[B48] Boldin MP Goncharov TM Goltsev YV Wallach D Involvement of MACH, a novel MORT1/FADD-interacting protease, in Fas/APO-1- and TNF receptor-induced cell death Cell 1996 85 6 803 815 10.1016/S0092-8674(00)81265-9 8681376

[B49] Benchoua A Couriaud C Guegan C Tartier L Couvert P Friocourt G Chelly J Menissier-de Murcia J Onteniente B Active caspase-8 translocates into the nucleus of apoptotic cells to inactivate poly(ADP-ribose) polymerase-2 J Biol Chem 2002 277 37 34217 34222 10.1074/jbc.M203941200 12065591

[B50] Cursi S Rufini A Stagni V Condo I Matafora V Bachi A Bonifazi AP Coppola L Superti-Furga G Testi R Src kinase phosphorylates Caspase-8 on Tyr380: a novel mechanism of apoptosis suppression EMBO J 2006 25 9 1895 1905 10.1038/sj.emboj.7601085 16619028 PMC1456929

[B51] Barbero S Barila D Mielgo A Stagni V Clair K Stupack D Identification of a critical tyrosine residue in caspase 8 that promotes cell migration J Biol Chem 2008 283 19 13031 13034 10.1074/jbc.M800549200 18216014 PMC2442311

[B52] Barbero S Mielgo A Torres V Teitz T Shields DJ Mikolon D Bogyo M Barila D Lahti JM Schlaepfer D Caspase-8 association with the focal adhesion complex promotes tumor cell migration and metastasis Cancer Res 2009 69 9 3755 3763 10.1158/0008-5472.CAN-08-3937 19383910 PMC2684981

[B53] Besnault-Mascard L Leprince C Auffredou MT Meunier B Bourgeade MF Camonis J Lorenzo HK Vazquez A Caspase-8 sumoylation is associated with nuclear localization Oncogene 2005 24 20 3268 3273 10.1038/sj.onc.1208448 15782135

[B54] Guo WH Yuan LH Xiao ZH Liu D Zhang JX Overexpression of SUMO-1 in hepatocellular carcinoma: a latent target for diagnosis and therapy of hepatoma J Cancer Res Clin Oncol 2011 137 3 533 541 10.1007/s00432-010-0920-x 20502916 PMC11957374

[B55] Jain S Singhal S Lee P Xu R Molecular genetics of hepatocellular neoplasia Am J Transl Res 2010 2 1 105 118 20182587 PMC2826827

[B56] Lee JS Thorgeirsson SS Genome-scale profiling of gene expression in hepatocellular carcinoma: classification, survival prediction, and identification of therapeutic targets Gastroenterology 2004 127 5 Suppl 1 S51 S55 15508103 10.1053/j.gastro.2004.09.015

